# How Do Cortical Excitatory Neurons Terminate Their Migration at the Right Place? Critical Roles of Environmental Elements

**DOI:** 10.3389/fcell.2020.596708

**Published:** 2020-10-23

**Authors:** Yumiko Hatanaka, Tatsumi Hirata

**Affiliations:** ^1^Graduate School of Frontier Biosciences, Osaka University, Suita, Japan; ^2^Brain Function Laboratory, National Institute of Genetics, Mishima, Japan; ^3^Department of Genetics, Graduate School of Life Sciences, Graduate University for Advanced Studies (SOKENDAI), Mishima, Japan

**Keywords:** cell adhesion, layer formation in the neocortex, marginal zone, radial glial cell, radial migration

## Abstract

Interactions between neurons and their environment are crucial for proper termination of neuronal migration during brain development. In this review, we first introduce the migration behavior of cortical excitatory neurons from neurogenesis to migration termination, focusing on morphological and behavioral changes. We then describe possible requirements for environmental elements, including extracellular matrix proteins and Cajal–Retzius cells in the marginal zone, radial glial cells, and neighboring neurons, to ensure proper migration termination of these neurons at their final destinations. The requirements appear to be highly linked to sequential and/or concurrent changes in adhesiveness of migrating neurons and their surroundings, which allow the neurons to reach their final positions, detach from substrates, and establish stable laminar structures.

## Introduction

The cerebral cortex is critical for memory formation, language, perception, attention, and other intellectual activities. These functions are supported by six layered neuronal structures, which are composed of excitatory and inhibitory neurons. The former account for about 80% of neurons in the cerebral cortex and transmit signals over long distances, projecting to multiple cortical areas as well as subcortical regions.

Neuronal migration is one of the most fundamental processes for constructing functional brain circuits in development. In the cerebral cortex, excitatory neurons are born in the ventricular zone (VZ) facing the ventricle and migrate toward their final positions, where they form a specific layered structure. Their aberrant migration and consequent mispositioning result in structural and functional abnormality, which underlies neuronal disorders such as epilepsy and intellectual disability (Romero et al., 2018).

Among the several stages in neuronal migration, termination of migration is the final important step and is directly associated with the establishment of the cortical cytoarchitecture. However, our knowledge about how neurons terminate their migration is still limited. Although this event must ultimately be analyzed *in situ*, most studies so far have been carried out in organotypic brain slice cultures. In such preparations, it is not easy to preserve intact radial glial (RG) cells that maintain the contact between their fibers and meninges, which is required for recapitulating proper termination of migration. The analysis also involves technical limitations in gene manipulation: *in utero* electroporation or viral infection to introduce a gene of interest usually targets neural stem cells, which can sometimes prevent us from examining the gene’s role in migration or migration termination when the transgene severely impairs neuronal differentiation and/or neuronal migration in the early phase. Nevertheless, recent studies using conditional knockout mice, or temporally and/or spatially controlled gene manipulation, are increasingly uncovering the process of migration termination, with particular attention being directed to sequential changes in adhesiveness between a migrating neuron and the extracellular components, including neighboring neurons, in its environment (Franco et al., 2011; Sekine et al., 2011, 2012; Gil-Sanz et al., 2013; Kohno et al., 2015; Ha et al., 2017; Matsunaga et al., 2017; Hirota et al., 2018; Hatanaka et al., 2019; Hirota and Nakajima, 2020).

In this review, we focus on the terminal phase of neuronal migration and discuss the role of these environmental components including extracellular matrix proteins and Cajal–Retzius (CR) cells in the marginal zone (MZ), RG cells, and neighboring neurons. Their cooperation is indispensable for proper migration termination, and thus for the construction of the proper cortical laminar structure.

## Migration Behavior of Cortical Excitatory Neurons

The development of cortical excitatory neurons from their progenitors is well documented. They are derived from neural stem cells in the cortical VZ through interkinetic nuclear migration, a cell cycle-dependent periodic movement of the nuclei. Initially, they divide symmetrically to amplify self-renewing stem cells. These cells have a bipolar morphology, extending apical and basal processes that are attached to the ventricular surface and the pia matter, respectively. They then further elongate their basal process and are called RG cells from around this stage, based on their molecular and morphological features (Malatesta et al., 2000).

**FIGURE 1:**
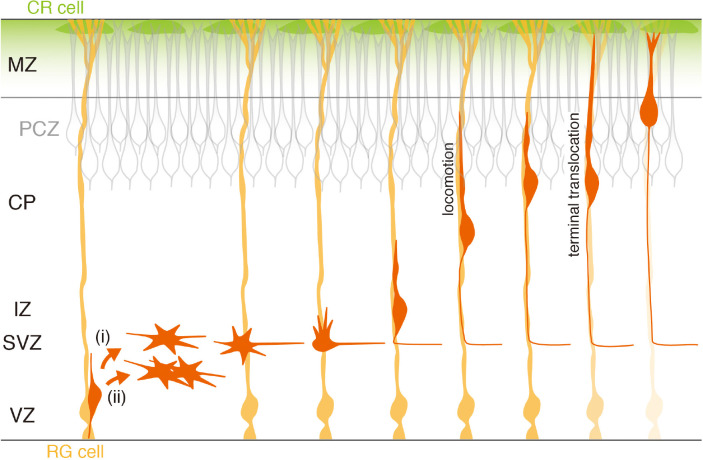
Neurogenesis, migration, and migration termination of excitatory cortical neurons. RG cells (orange) are neural progenitors as well as substrates for radially migrating neurons. They produce neurons (red) directly (i) or indirectly via intermediate neuronal progenitors (ii). Newly generated neurons reside transiently in the SVZ/IZ as multipolar neurons, and initiate axon formation. After forming a leading process, they start radial migration along RG fibers, leaving the elongating axon in the rear. They initially migrate in locomotion mode but finally change to terminal translocation mode by anchoring the leading process to the marginal zone (MZ). Underneath the MZ, postmigratory neurons are densely packed, forming the primitive cortical zone (PCZ) (Sekine et al., 2011). The mode change likely allows newly arrived neurons to integrate into the PCZ. Neuron–RG cell adhesion disappears during the terminal phase of migration (as indicated by the fading RG color).

In the neurogenic period, an RG cell divides asymmetrically, producing two daughter cells: one of them remains an RG cell, while the other differentiates into either a neuron or an intermediate neuronal progenitor (IP) (Haubensak et al., 2004; Miyata et al., 2004; Noctor et al., 2004; Figure 1). Both neurons and IPs migrate toward the sub-VZ (SVZ), retracting their apical and basal processes (Tabata and Nakajima, 2003). They lose apicobasal polarity and execute “multipolar migration,” alternately extending and retracting thin and short processes, and gradually move into the intermediate zone (IZ). IPs further divide to produce two or more daughter neurons during this period.

These daughter neurons in the IZ suddenly begin to elongate a dynamically moving short process, which often eventually becomes an axon (Hatanaka and Yamauchi, 2013; Namba et al., 2014). After forming pia-directed thick leading processes, they initiate radial migration toward the pial surface. Migrating neurons, which are closely apposed to RG fibers (Rakic, 1971, 1972), show a bipolar shape, extending a leading process in front and a long trailing process, a nascent axon, at the rear (Hatanaka and Yamauchi, 2013). They show repeated saltatory movements, termed “locomotion” (Nadarajah et al., 2001): typically, they first extend a leading process forward up to a certain length away from the soma, and then the leading process becomes anchored, followed by a forward movement of the soma. Although the dynamic movement of the leading process does not always appear to be strictly coupled with that of the soma (Schaar and McConnell, 2005), their overall combined behavior results in saltatory movement. When these neurons approach the top of the cortical plate (CP), they appear to change their migration mode. After the leading process reaches the MZ, their somas pause transiently and then move quickly along the shortening leading process, which is called “terminal translocation” (Nadarajah et al., 2001; Sekine et al., 2011). Finally, the neurons settle at the top of the CP. Since later-born neurons migrate past the neurons in existing layers before terminating their own migration, these sequential neuronal migratory behaviors lead to the establishment of a cortical laminar structure that exhibits an “inside-out” organization (Angevine and Sidman, 1961; Rakic, 1974).

## Roles of Matrix Proteins and CR Cells in the MZ During the Terminal Phase of Migration

As noted above, when migrating neurons approach the MZ, they change their mode of migration from locomotion to terminal translocation (Sekine et al., 2011). The first step of this change is to anchor their leading process to the MZ (Nadarajah et al., 2001; Sekine et al., 2011; Figure 1), which is a critical step toward migration termination (Sekine et al., 2011). Morphologically, when cortical neurons migrate radially in a deep part of the CP, they show the saltatory nuclear movement typical of locomotion. They then transiently pause when they approach the top of the CP, followed by rapid somal movement accompanied by shortening of the leading process, whose tip remains attached to the MZ (Sekine et al., 2011).

**FIGURE 2:**
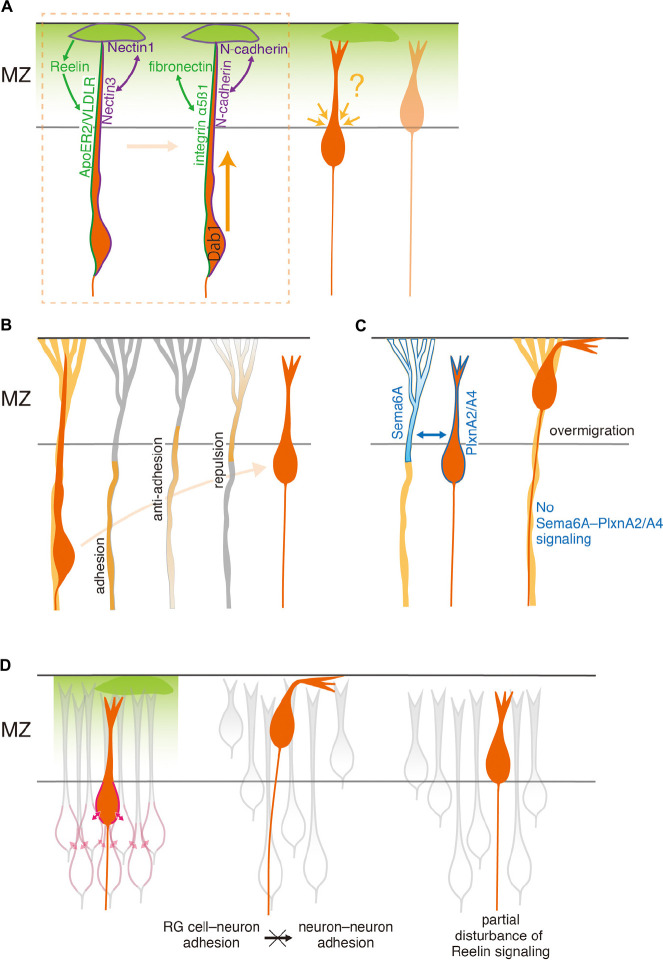
Cellular and structural elements required for proper migration termination. **(A)** Roles of extracellular matrix and CR cells in the MZ. Radially migrating neurons anchor their leading process to the MZ or to CR cells in the MZ, through contact-independent Reelin–receptor interaction as well as contact-dependent Nectin1–Nectin3 interaction (left cell in the area surrounded by a broken line). These interactions promote adhesion between fibronectin in the MZ and integrin α_5_ß_1_ on the neuron, and by homophilic N-cadherin adhesion between CR cells and neurons, respectively (right cell). Dab1 is essential for executing terminal translocation. Although the MZ is important for the terminal translocation, it remains unsolved whether it also contributes to determining the position of the soma, which never invades the MZ. **(B)** Roles of RG cells. Radially migrating neurons detach from the RG cell fiber at the distal part. This detachment likely occurs by a decrease of adhesion, increase of anti-adhesion, and/or increase of repulsion between RG cells and neurons. **(C)** Sema6A on RG cells and PlxnA2/A4 on migrating SLNs appear to work as a repulsion signal that detaches neurons from RG cells. The absence of a Sema6A–PlxnA2/A4 signaling results in ectopic SLNs positioned beyond their proper final destination, likely due to the lack of detachment. **(D)** Roles of neighboring neurons. Radially migrating neurons are stably settled at the final position. This may be achieved by increased neuron–neuron adhesion (left). Over-adhesion of neurons to RG cells may reduce neuron–neuron adhesions, and lead to loosely packed neurons in the CP and ectopically located neurons in the MZ (middle). Direct inhibition of neuron–neuron adhesion, which may be induced by reduction of Reelin signaling, also causes loosely packed neurons and neurons ectopically located in the MZ (right).

Several studies have indicated that Disabled homolog 1 (Dab1) plays a critical role in terminal translocation (Olson et al., 2006; Franco et al., 2011; Sekine et al., 2011), molecularly accounting for this mode change (Figure 2A). Dab1 is an intracellular adaptor protein that transduces signaling of Reelin (Rice et al., 1998; Howell et al., 1999), an extracellular matrix protein synthesized by CR cells. Two receptors of Reelin, apolipoprotein E receptor 2 (ApoER2, also known as LRP8) and very low-density lipoprotein receptor (VLDLR) (D’Arcangelo et al., 1999; Hiesberger et al., 1999; Trommsdorff et al., 1999), are expressed on the leading process of migrating neurons that extend into the MZ (Hirota et al., 2015). When Dab1 in cortical neurons destined for layers 2/3 is suppressed by RNA interference (Olson et al., 2006; Sekine et al., 2011) or knocked out (Franco et al., 2011), the neurons approach the top of the CP but fail to reach their final positions. These findings support the notion that Dab1 is required for the terminal translocation of cortical neurons.

Stable attachment of the leading process to the MZ may be a key for terminal translocation. Indeed, Dab1-deficient neurons extend the leading process into the MZ but fail to maintain contact with the MZ. They often retract the process and rarely undergo terminal translocation (Franco et al., 2011). Consistent with this, although upper-layer neurons whose Dab1 is knocked down extend the tip of the leading process into the MZ, the process is underdeveloped and less likely to contact the MZ (Olson et al., 2006). The attachment is likely mediated by a Reelin–Dab1–Crk/CrkL–C3G–Rap1 pathway that activates α_5_ß_1_ integrin on neurons, which promotes neuronal adhesion to fibronectin in the MZ (Sekine et al., 2012). However, deletion of ß_1_ integrin from neurons does not cause major defects in brain lamination (Belvindrah et al., 2007; see also next section), suggesting that fibronectin binding via α_5_ß_1_ integrin activation is dispensable for terminal translocation. Homophilic N-cadherin adhesion between leading processes and CR cells via the Rap1 pathway also seems to function (Franco et al., 2011) (see also below).

The cell-dense outermost part of the CP is named the primitive cortical zone (PCZ), which is occupied by newly settled immature neurons (Sekine et al., 2011; Figure 1). The terminal translocation step brings somas of migrating neurons into the PCZ, where inside-out placement of neurons occurs (Sekine et al., 2011). Indeed, sequential labeling of VZ cells at embryonic day (E)14.5 and 15.5 with different fluorescent proteins shows clear birthdate-dependent inside-out alignment of neurons in the wild-type cortex. However, when Dab1 in VZ cells at E15.5 is suppressed, these labeled neurons are not segregated at the top of the CP, failing to form the inside-out layer pattern. Thus, terminal translocation appears to be critical for migrating neurons to properly position their somas within the PCZ to establish the inside-out alignment.

Adhesion molecules expressed by CR cells also appear to play a critical role in terminal translocation. Gil-Sanz et al. (2013) showed that CR cells express an immunoglobulin-like cell adhesion molecule, Nectin1, while the leading processes of migrating cortical neurons express its preferred binding partner, Nectin3 (Figure 2A). Knockdown of either of these genes causes a failure of terminal translocation. The binding of Nectin1 to Nectin3 in migrating neurons stabilizes homophilic N-cadherin interactions between neurons and CR cells. This is mediated by the recruitment of an Afadin/Rap1 complex, an essential regulator of cadherin function via p120 Catenin, to the Nectin1–Nectin3 contact site (Gil-Sanz et al., 2013). Disturbance of N-cadherin function in CR cells or migrating cells leads to impairment of the leading process, which then displays reduced arborization in the MZ, and failure of somal translocation (Gil-Sanz et al., 2013). Interestingly, Reelin promotes recruitment of p120 Catenin and N-cadherin to Nectin/Afadin complexes, thereby stabilizing N-cadherin so that it can mediate homophilic interactions at the cell surface. Thus, cooperation between secreted and contact-dependent signals from CR cells may be essential for terminal translocation.

While leading processes are anchored to the MZ, migrating neurons arrest somal movement just beneath the MZ, forming a sharp boundary between the CP and the MZ. Therefore, there must be mechanisms that regulate the somal movement. So far, however, there is no evidence for direct regulation of somal movement by the MZ. Reelin signaling possibly contributes indirectly to this process through enhancement of neuron–neuron interactions at the top of the CP, but we need further studies to test this hypothesis (see also section “Roles of neighboring neurons during the terminal phase of migration”).

## Roles of RG Cells During the Terminal Phase of Migration

Radially migrating neurons migrate along RG fibers. During this mode of migration, they maintain specific adhesive interactions with RG cells, indicating the importance of these interactions for the migration. A special junction termed “interstitial density” is observed between actively migrating, but not stationary, neurons apposed to glial fibers (Gregory et al., 1988). When these neurons enter the terminal phase of migration, their specific adhesive interactions with RG cells are presumed to be dissolved. This could occur before the terminal translocation (Nadarajah et al., 2001) or concurrently with the neurons settling in their final destinations. Studies including previous *in vitro* culture analyses as well as recent gene-manipulated mouse experiments have uncovered several molecules that are involved in these processes (Anton et al., 1996, 1999; Gongidi et al., 2004; Hatanaka et al., 2019).

Anton et al. (1996, 1999) first reported molecules that might mediate adhesive interactions between neurons and RG cells. These include RG cell membrane proteins, recognized by specific antibodies (Anton et al., 1996), and α_3_ß_1_ integrins (Anton et al., 1999). Gap junction subunits connexin 26 and connexin 43 may also mediate adhesion, since they are expressed at the contact sites between migrating neurons and RG fibers (Elias et al., 2007). These molecules contribute to the continuation of migration. Importantly, the membrane proteins, localized at the plasmalemmal junction between migrating neurons and RG fibers, are distributed along the RG fibers but are virtually absent in their distal part that resides within the MZ (Anton et al., 1996). Moreover, functionally blocking the molecules perturbs migration, sometimes leading to detachment of neurons from their RG fiber substrates *in vitro* (Anton et al., 1996, 1999) and *in vivo* (Elias et al., 2007). Although the distribution of these molecules has not been fully reported, these findings suggest that proper migration termination depends on the spatial distribution of RG adhesive molecules, and that a reduction of such molecules on RG fibers causes premature termination of neuronal migration (Figure 2B). However, the identity of the membrane antigen remains unknown. Furthermore, later studies raised a question regarding the direct contribution of integrins to neuron–RG cell adhesion, because removal of ß1 integrin from RG cells, but not from neurons, perturbs layer formation, accompanied by disruption of endfeet anchorage on the pial basement membrane (Graus-Porta et al., 2001; Belvindrah et al., 2007). Thus, it is possible that integrin functions indirectly in neuronal migration *in vivo* by maintaining the integrity of the pial basement membrane (Graus-Porta et al., 2001; Halfter et al., 2002; Belvindrah et al., 2007).

There is another type of RG surface protein, SPARC (secreted protein acidic and rich in cysteine)-like 1, which is contrastingly expressed in the distal segment of the RG fibers spanning the upper CP. Its spatial expression profile and anti-adhesive activity between neurons and RG cells in culture suggest that it functions as a trigger for migrating neurons to detach from RG cells at their final positions (Gongidi et al., 2004; Figure 2B). Consistently, mutant mice lacking this molecule exhibit diffuse laminar organization. However, their gross cortical organization is normal, suggesting that other molecules are also involved in terminating migration. Molecules that interact with SPARC-like 1 remain unknown, and a better understanding of the active detachment process is awaited.

As a novel molecular cue, we recently found that a Semaphorin (Sema) 6A–Plexin (Plxn)A2/A4 interaction is responsible for the detachment of migrating neurons from RG fibers (Hatanaka et al., 2019; Figures 2B,C). Sema–Plxn interactions were originally determined as repulsive signals in axonal guidance (Tamagnone and Comoglio, 2000). Either Sema6A single mutants or PlxnA2/A4 double mutants show mislocalization of superficial layer neurons (SLNs) in the MZ, as the result of overmigration of SLNs beyond their final destinations. Sema6A is expressed in RG cells, while PlxnA2 and A4 are predominantly expressed in SLNs at the time when they terminate migration. Conditional knockout of Sema6A in RG cells recapitulates the overmigration phenotype, while forced expression of PlxnA2 in SLNs rescues the phenotype of PlxnA2/A4 double mutants, indicating that Sema6A and PlxnA2/A4 function in RG cells and neurons, respectively. Since Sema6A–PlxnA2/A4 trans-interaction typically elicits a repulsive effect, it is very likely that interaction between Sema6A on RG cells and PlxnA2/A4 on SLNs terminates neuronal migration by detaching SLNs from their RG substrates at their final destinations. Consistent with this interpretation, the extracellular domain of PlxnA2 most strongly binds to the MZ, highlighting their potential interacting site (Hatanaka et al., 2019). These results support the idea that active changes in adhesiveness, or a repulsive interaction, between neurons and RG cells function for proper termination of radial migration.

Terminal translocation is often referred to as an RG cell-independent process (Nadarajah et al., 2001), which implies that the detachment from RG cells itself is somewhat coupled with the terminal translocation. Supporting this notion, for example, Dab1 signaling is implicated in regulating the de-adhesion from RG cells; radially migrating neurons in *Dab1* deficient mice remain closely attached to the process of parental RG cells, but they detach from the process when they are forced to express wild-type Dab1, but not Dab1 mutants that lack potential phosphorylation sites (Sanada et al., 2004). The Nectin3–Nectin1 interaction also seems to contribute to the detachment process; their interaction switches N-cadherin-mediated neuronal adhesion from RG cells (Kawauchi et al., 2010) to CR cells (Gil-Sanz et al., 2013). In addition, the detachment from RG cells can play a role in stopping somal movement at the top of the CP by removing the migration substrate. Therefore, in addition to identifying the molecules involved in the detachment, determining the precise timing of neurons’ detachment from the RG substrate, in relation to the terminal translocation, is an essential piece of information to understand the mechanisms of migration termination.

## Roles of Neighboring Neurons During the Terminal Phase of Migration

It is conceivable that cortical neurons arriving at their final destinations preferentially adhere to each other, allowing them to make laminar structures (Goffinet, 1984; Figure 2D). Indeed, the formation of the PCZ, where neurons are tightly packed underneath the MZ, suggests an increase of postmigratory neuron–neuron adhesiveness (Sekine et al., 2011). A PCZ-like structure is observed not only at late stages but also at earlier stages when numerous cortical neurons are migrating (Catalano et al., 1991). Therefore, all radially migrating neurons, when they reach the top of the CP, encounter a wall of cells compacted by neuron–neuron homophilic interactions. It is also hypothesized that the increase of adhesiveness among neurons helps detachment of neurons from the RG substrate by counteracting neuron–RG interaction (Goffinet, 1984).

Theoretically, the switch in adhesiveness of migrating neurons from RG cells to neighboring neurons can be achieved by either weakening of the neuron–RG cell interaction or strengthening of the neuron–neuron interaction. Several studies suggest that both occur, not independently but cooperatively or sequentially. As described above, the molecules that are responsible for migrating neuron–RG cell adhesion include integrins (Anton et al., 1999). Interestingly, while α_V_ integrin is important to maintain optimal neuron–RG cell adhesive strength, α_3_ integrin appears to modulate neuron–RG recognition cues; impairment of α_3_ integrin function switches adhesive preference of neurons from gliophilic to neurophilic in dissociated cell culture (Anton et al., 1999). Thus, it is possible that a decrease of α_3_ integrin expression in the upper part of the CP weakens neuron–RG cell interactions and conversely strengthens neuron–neuron interactions.

Another example is observed in Sema6A–PlxnA2/A4 signaling-deficient mice, in which SLNs are less densely packed in the PCZ compared with those in wild-type mice at the stage when they reach their final position (Hatanaka et al., 2019). When the overmigration of SLNs in PlxnA2/A4 double mutant mice was rescued by forced expression of PlxnA2, these neurons were also clustered densely, possibly because of weakened neuron–RG cell interactions. These observations suggest that weakened neuron–RG cell interactions lead to an increase of neuron–neuron interaction, implying that they are interconnected events.

Interestingly, Reelin appears to play a direct role in the increase of neuron–neuron interaction. Matsunaga et al. (2017) have found that application of Reelin to dissociated cortical neurons transiently enhances neuronal adhesion. This is consistent with the finding that forced Reelin expression in migrating neurons induces neuronal aggregation (Kubo et al., 2010). Although a low level of Reelin expressed in the lower IZ (Uchida et al., 2009; Hirota et al., 2015) is reported to have a different role for migrating multipolar neurons in the IZ (initiation of the multipolar–bipolar transition/control of neuron–RG cell interaction, Jossin, 2011; Jossin and Cooper, 2011; Kon et al., 2019), accumulation of these neurons in the lower IZ (Tabata et al., 2009) may also support this view. The increase of neuron–neuron interaction seems to be N-cadherin-dependent (Matsunaga et al., 2017). In this context, a Reelin–receptor–Dab1 pathway likely functions, because the aggregation fails to occur when binding of Reelin to the receptor is prevented by 2A-Reelin, or when Dab1 is removed from the system. *In vivo*, neurons are mislocalized in the MZ of mice that partially lack Reelin signaling, such as the single-gene deletion of Reelin receptor components (VLDLR, Hack et al., 2007; Hirota and Nakajima, 2020, and ApoER2, Hirota et al., 2018), and of Reelin mutants that lack the C-terminal region (Kohno et al., 2015; Ha et al., 2017). Moreover, forced Reelin expression in migrating neurons in these mutant mice indicates a requirement of these receptors for the formation of properly packed PCZ-like aggregates (Hirota et al., 2018; Hirota and Nakajima, 2020). Collectively, these observations reveal that it is highly likely that Reelin–ApoER2/VLDLR receptor signaling controls neuron–neuron adhesions. Thus, a likely scenario is that Reelin secreted from CR cells controls the neuron–neuron adhesions during the terminal phase of migration, thereby indirectly suppressing ectopic neuronal invasion of the MZ, and eventually assures the stable settlement of newly arriving neurons at the top of the CP.

Dab1 stability appears to be important as a cell-autonomous determinant of neuronal positioning. Knockdown of Cullin-5 (Cul5), a key component of the E3 ubiquitin ligase complex, prevents the Reelin-dependent degradation of phosphorylated Dab1, causing activated Dab1 to accumulate in migrating neurons (Feng et al., 2007; Simo et al., 2010). These neurons are positioned more superficially, suggesting their overmigration. If activated Dab1 induces neuron–neuron adhesion to terminate migration, overmigration caused by Dab1 activation would appear to be contradictory. This seeming discrepancy may not arise, however, given that Dab1 is normally degraded upon Reelin stimulation (Arnaud et al., 2003). Moreover, the Cul5-knocked down neurons show an increase in migration speed as well as persistence at the top of the CP (Simo et al., 2010). These observations suggest that temporally regulated activation and degradation of Dab1 normally occur *in vivo*, thereby effecting transient strengthening and weakening in neuron–neuron adhesion at the top of the CP to terminate migration.

Finally, we would like to discuss the phenotypic similarity between mutant mice that have primary defects in different cellular contexts. As described above, a decrease of Reelin signaling leads to mislocation of SLNs in the MZ (Hack et al., 2007; Kohno et al., 2015; Ha et al., 2017; Hirota et al., 2018; Hirota and Nakajima, 2020), which is reminiscent of the phenotype observed in mice lacking Sema6A–PlxnA2/A4 signaling (Hatanaka et al., 2019). However, unlike Sema6A–PlxnA2/A4 knockout mice that display impaired neuron–RG cell interactions, the mutation in Reelin signaling appears to cause changes in neuron–neuron interactions, because none of VLDLR, ApoER2, or Reelin appear to be expressed by RG cells at the stage when SLNs reach the top of the CP (Alcantara et al., 1998; Hirota et al., 2015). Although it is still possible that Reelin signaling affects neuronal migration through control of the RG scaffold (Hartfuss et al., 2003; Chai et al., 2015), the above observation raises the possibility that neuron–RG cell and neuron–neuron interactions are interrelated processes in the proper location of neurons in cortical layers.

## Concluding Remarks

Recent studies illuminate the roles of environmental elements in migration termination and proper positioning of cortical excitatory neurons. These include extracellular matrix proteins and CR cells in the MZ, RG cells, and neighboring neurons. Although their roles are not completely separable, each element appears to directly control the contiguous processes of terminal translocation, neuronal positioning, and proper alignment of newly arrived neurons at the top of the CP. Migrating neurons dynamically change their adhesiveness to these elements during the terminal phase of migration. Importantly, changes in adhesiveness are cooperatively regulated by these different elements, so that migrating neurons can sequentially switch their adhesion during the terminal phase of migration. This is achieved by multiple signaling molecules, such as Reelin and N-cadherin, that control the strength of cell adhesion, as well as adhesion-related molecules that regulate adhesion specificity between a neuron and each element.

Because migration termination is a highly dynamic process, understanding such a process will require dynamic analyses of adhesion-related molecules within cells as well as between cells in live-cell imaging, utilizing techniques such as SLENDR that clarifies the localization of intrinsic proteins (Mikuni et al., 2016), pHluorins to monitor protein surface expression (Miesenbock et al., 1998; Ashby et al., 2004), and FRET (Förster resonance energy transfer) biosensors to detect downstream intracellular signaling (Nakamura et al., 2006; Pertz et al., 2006) in a spatiotemporal context. Also, we will need to identify molecules that are directly involved in adhesion, as well as those that serve to switch adhesiveness during migration termination, and to analyze them in a temporally and spatially controlled manner.

## Author Contributions

YH wrote the manuscript in consultation with TH. Both authors contributed to the article and approved the submitted version.

## Conflict of Interest

The authors declare that the research was conducted in the absence of any commercial or financial relationships that could be construed as a potential conflict of interest.
